# A Rare and Instructive Disease of the Ear

**Published:** 1873-02

**Authors:** A. W. Calhoun

**Affiliations:** Vienna, Austria


### FOREIGN CORRESPONDENCE.

### A RARE AND INSTRUCTIVE DISEASE OF THE EAR.

BY A. W. CALHOUN, M.D.

I venture the assertion that seldom, if at all, has it occurred during the practice of most physicians to have met with a case of aural disease produced, or at any rate intensified, by the presence in the ear of what is vulgarly, yet properly, called maggots. It must be admitted that the existence of such animals in the human ear is of so great a rarity as to be well-calculated to originate a doubt in the minds of many as to *any* contingency occurring sufficient to give cause to the birth of such a loathsome affection in which maggots play the chief roll. While attending the clinics of Prof. Gruber, one of these cases presented itself, which so interested me with its many peculiarities that I followed it during many weeks, and watched its progress from day to day till complete recovery; and it being thus comparatively unique, and at the same time very instructive as well as intensely interesting, I am induced to give its entire history, progress, etc., believing it to contain also something of interest for many others, although affections of the ear may not be immediately or directly occupying their attention.

On the 17th June, 1872, H. S., a man aged thirty-five, presented himself at the clinic of Prof. Gruber for the relief of the intolerable pain in his right ear. He was at that moment suffering, and upon being questioned as to the cause, etc., the following history was communicated: Four days previously, on the 13th, he received a severe blow upon the right side of face and head, from a lamp thrown by a man with whom he was in quarrel. The immediate effects of the blow were nothing more than a cut upon the side of the head and profuse hemorrhage, but nothing unusual about the ear was noticed. On the night of the same day he slept in a horse stall, and during the following morning felt slight pain and a continuous buzzing in the right ear, both of which, in conjunction with giddiness, so increased in intensity as to force him to consult a physician on the succeeding day, who, finding the ear and its vicinity besmeared with coagulated blood, much swollen and extremely painful to the touch, and not venturing to treat such an aggravated case, sent his patient to the above named clinic. Such was his condition on entering, his face being pale and indicative of much suffering. After removing the encrusted blood and examining the interior of the ear, the first glance revealed a quantity of pus filled with a moving, living mass, whose occasionally sudden movements gave the patient such agony as to cause him to spring from his chair with a cry of pain. By repeated and vigorous syringing with milk-warm water, the pus was emptied, and finally, but after much trouble, were also removed five (5) large half inch long, living maggots, which, taking into consideration their size, activity and stubborness, were apparently capable of producing even more serious and permanent damage than they had as yet inflicted upon their victim. It was a real gratification to mark the instantaneous relief of the patient, for no sooner was the last of these troublesome inhabitants of the ear brought into the outside world, than he expressed himself thankfully for his rapid transition from torture to comparative ease. A further examination showed the membrana tympani much inflamed and enormously swollen, the outer surface rising and sinking into wave-like folds. The great sensitiveness of the parts prevented a minute investigation, but, as far as could be observed, no perforation had yet taken place. As an astringent, and to ward off a return of any violent pain, the following solution was ordered: ℞.—Plumbi acetat., morphiæ sulph. aa gr. j, aquæ distill. ℥i. Several drops of which to be dropped into the ear three or four times daily.

June 18th.—During the night the pain returned, and was not confined alone to the ear but extending over the whole head, and this with the buzzing, which had increased to an immense roaring, deprived the patient of sleep or rest. Since morning these symptons have somewhat subsided, he being now in tolerable ease. The membrana tympani is still inflamed, but not so swollen as yesterday, the handle of the malleus, with its processus brevis, being brought into view by using a strong light, and in the anterior inferior periphery a small oval, pinhead sized perforation is discovered, which very probably existed also yesterday, but was concealed by the overlapping swollen cutis on and about the membrana tympani. Surrounding the perforation is a white circular exudation, and in the light reflecting triangle of the membrane a clearly visible pulsation which, in most instances, is an evidence of a continuation of a violent inflammation, and also evidence either of the present existence of a perforation or that such is at least threatening to take place. After syringing the ear and ridding it of pus, and using the air douche by means of a catheter introduced into the eustachian tube and throwing air through it by compressing a common rubber balloon, the perforation was more evident, the air passing out through the opening, forcing away from its edges the thick and dry secretion which had given it the apparently small size as at first seen. In this case, as in all others, the passage of a stream of air from the eustachain tube through the perforation into the external ear, produced that characteristic sharp, whistling sound, which is so often of importance to us in a diagnostic point of view, in so far that when this whistling or hissing noise ("Perforations Geraensch" of the Germans) is heard, we can be positively assured that a perforation of the membrana tympani exists at some point, though we may have for hours sought in vain for it with the eye, and with other means we may have at our disposal.

June 20th.—The membrana tympani remains the same in regard to its inflammatory symptoms, nor has the perforation increased any in size, but is as yesterday. The pain and buzzing, or singing, in the interior of the ear, is not so annoying, occurring in paroxysms with more or less extended intervals of complete freedom. A continual and copious suppurative secretion from the deeper parts demands constant care and attention, as much of the cure depends upon the unimpeded outward flow of the pus, and, as far as possible, upon extreme cleanliness of the ear, both internally and externally. The parts before the tragus are to-day much swollen, and, together with the walls of the external meatus, very sensitive to the slightest touch, demonstrative of an extension of the inflammation along the walls from the inner to the outer and superficial structures. His hearing is materially affected, both for the watch and speech—the ordinary modes for trying the hearing distance. With the unaffected ear firmly closed, the ticking of a large sized pocket watch is perceived only when brought within one inch of the diseased ear, and single words, spoken in the usual conversational tone, can be scarcely distinguished at a distance of a few feet. After syringing and using the air douche, the hearing remains unchanged, which is unexpected and unusual, since mostly by such treatment, especially the latter, the hearing is considerably, and often surprisingly, improved, unless some lesion has already taken place affecting the vibrations of the ossicles or the perception of the acousticus.

To reduce the inflammation and prevent a further extension of the external swelling, two (2) leeches are applied half an inch before the tragus, with the view of drawing a greater or less quantity of blood. The previous treatment is also continued.

June 21st.—Through the agency of the leeches the external swelling is to a great extent reduced, and many of the inflammatory symptoms have disappeared, so that the parts are less painful or sensitive, and even the troublesome and disagreeable singing noises in the ear have greatly diminished in violence and frequency. The patient having slept without disturbance during the whole of the previous night, and having regained, and, for the present at least, satisfied his appetite, is to-day more communicative about the origin of his trouble, and its progress up to the time he entered the clinic, and amongst other things, we learn that before entering, he himself picked two (2) maggots from his ear, making in all seven (7.) Several small abscesses are observed forming in the meatus externus and on the soft parts externally, but these give the patient no concern, and are, in fact, of but little consequence, being quite small and painless. A continuation of the previous treatment.

June 26th.—With the exception of the slight pain in the inflamed parts in front of the ear, the patient makes no complaint whatever, the pain from the now less inflamed and less swollen membrana tympani having completely vanished, leaving but little of the former extremely unpleasant buzzing or singing. The pulsations on the membrana tympani are still visible, showing that the inflammation continues to be of importance, and a further proof of this is the constant otorrhœa, which, although having been considerably benefited by the applied remedies, is yet quite copious. The perforation has possibly diminished in size to a very small extent, the opposing sides approaching nearer each other by the growth of granulations upon the edges.

July 1st.—Nothwithstanding the attentive use of all the means before mentioned, the otorrhœa has undergone no great improvement—the secretion flowing almost as freely from the ear at present as at any time previous. In all other respects, however, the general appearance of the diseased organs, as well as the declarations of the patient, give us a striking example of the rapidity with which acute aural affections improve when promptly taken in hand. Neither pain nor giddiness, nor the deep-seated roaring, call for any attention; even the perforation grows day by day perceptibly smaller, healing almost of its own accord since the inflammation of the adjacent parts has been reduced, leaving behind only the otorrhœa as a special charge, for which the following solution, instead of the acetat plumbi, which appears insufficient, is prescribed, viz: ℞.—Zinci sulph. gr. j; glycerine, aquæ distill. aa ℥ ss. A few drops milk-warmed to be poured into the ear several times daily.

July 12th.—During this interval, the patient has been treated as before mentioned, by careful cleanliness of the ear, the air douche, and the regular use of the astringent, and to-day we find all traces of the otorrhœa entirely disappeared, and excepting an occasional return of the buzzing or roaring, no unpleasant reminder of the former occupants of his ear exists. The perforation of the membrana tympani has been closed by a transparent cicatrice whose movements can be clearly traced by using the air douche, the remaining portion of the membrane has assumed its natural ashy gray color, and its proper thickness, the limits between the cicatrix and the uninjured membrane being distinctly defined. Also, has the inflammation and swelling in the meatus externus and the soft parts before and around the tragus vanished; in short, the patient is cured, with no other disadvantage than that in his membrana tympani a moderately large cicatrice has taken the place of the original sound membrane, producing, however, but little difficulty since his hearing now, both for the watch and speech, is very nearly normal. With the direction to continue for some time the daily syringing, and the use of the zinc solution, and proper protection to the ear, and to return occasionally to the clinic for the purpose of receiving the air douche, the patient is discharged from treatment.

That such an affection of the ear belongs to the extremist rarity, may be inferred from the fact that in the entire practice of Prof. Grnber, comprising many thousand patients annually, this is the fifth that has as yet come under his notice. If I am not mistaken, with perhaps one or two exceptions, the literature bearing upon aural diseases fails notably in communications relating to this trouble, whether produced traumatically or otherwise. The origin of these parasites is readily explained. In the treatment of ordinary external wounds, we know what caution is often requisite to guard against such unwelcome consequences of the least want of cleanliness, which brings a multitude of flies depositing their larvas on or about the wounds, and therefrom are shortly afterwards generated the maggots. So, also, is it with the ear in a few of these observed cases, the flies being attracted by the accumulated filth, such as a long undisturbed collection of cerumen or a neglected otorrhœa, or, as in the above case, clotted blood within and around the ear, depositing their eggs upon and within the collections, and in an incredibly short time, often in a very few hours, developing into living, moving forms. In this special case, if the patient's history is to be believed (and it is by no means beyond belief,) not twenty-four hours had intervened between the reception of the blow and the removal by himself of two of the maggots.

Contrary to expectation, it is no easy matter to rid the ear of these animals, their resistance to removal and their tenacity to life being, in fact, wonderful. Usually, in such instances, a perforation of the membrana tympani has already ensued from a previous inflammation of whatever character, or quickly follows the violent inflammation brought about by the immediate presence and constant action of these parasites, giving them opportunity to pass through at will into the cavity of the tympanum. Upon the least disturbance from without, or by any attempt whatever at dislodgement, all, or as many as can, hurry intuitively, as it were, through the perforation, and firmly imbed their sharp beaks into the nooks and corners which may afford them protection behind the greater or less remnant of the membrana tympani. Here they defend themselves heroically, often resisting successfully, or at all events for a long while, the strongest injected streams of water, their bodies being thrown to and fro in all directions, and their bills being apparently fastened still deeper upon each injection, causing the patient a fresh paroxysm of acute pain. Where no perforation has yet taken place, they seek the deepest portion of the meatus externus and fasten themselves in the membrana tympani itself on or near its periphery, or near the slight circular groove formed by the insertion of the membrane into the surrounding bony or cartilaginous and fleshy parts. When thus situated, their stubbornness does not avail them so much as when (for themselves) more favorably located, viz: behind the membrane in the middle ear, but are more readily brought away by the use of the syringe as well as by other means.

In making some remarks upon this and the other few similar cases which had come under his observation, Professor Gruber narrated some experiments made by himself, demonstrating their invulnerability, so to speak, to medicaments which in most cases prove promptly destructive to both animal and vegetable parasitic life. In certain instances the ear was filled with spiritus terebinthinæ, and in others with undiluted alcohol, and still in others with other equally powerful fluids, and after an hour's residence in such a home, they were mostly to no great extent affected. In fact, more disadvantages seemed to have resulted to the patients themselves than to the animals, for, aside from the pain produced by the extremely long contact of such strong remedies with the inflamed and sensitive structures of the ear, a stimulus was apparently given the latter by being thus discommoded to take a deeper hold, contributing an additional cause for increased pain.

In the case above narrated, the maggots were removed after much trouble by using the syringe, they not having gone further than the lower part of the meatus externus; but this not unfrequently completely fails to move each and all from their moorings, a number contending successfully against the force of even a strongly-injected body of water. When this trouble is met with, the quickest and easiest way of removal is at once, with appropriately curved forceps and a good reflected light, to pick each one separately from its bed, thereby saving for yourself much time and patience, and saving the individual much unnecessary pain, given by the other various and continuous efforts.

It will be remembered that the patient, upon entering the clinic, complained of an intolerable buzzing or roaring and singing in his ear, which perhaps needs a few words of explanation. Tapping against or running the finger across a tightly-drawn head of a drum makes a well-known rough, rumbling noise. Upon somewhat the same principle of a drumhead is formed and situated the membrane constituting the outer wall of the tympanum, and hence its name—membrana tympani. Bearing these facts in mind, it is readily comprehended what a horrible sensation can be produced by the incessant boring and never-ceasing action of five or (as before entering the clinic) seven large half-inch long insatiable maggots against this delicate associate-conductor of sounds, the membrana tympani. As children, or at some period of our lives most of us, perhaps, have had the indescribably uncomfortable fright caused by the presence and movement in the ear of some kind of a harmless and equally frightened insect, imagine this ten-fold magnified as regards noise, and every other unpleasant feeling (setting aside the pain), and we may get some idea of what a patient experiences under the above circumstances, and also have one but not the only explanation of the roaring and singing in this instance. Were the presence of foreign substances in the external or even in the internal ear the prime cause of the "intolerable singing," and the often accompanying giddiness so frequently complained of by patients with ear affections, aural surgery would in a short while be freed from the severest opprobrium now resting over it—declaring its comparative inability to remove that long-continued, deep-seated, inveterate *singing* ("sausen"), unpleasantly known to all aurists. In our patient the membrana tympani, always rich in nerves and extremely sensitive, became enormously inflamed, and the irritation extending along the various nerve fibres from this point still further and further, and finally into the labyrinth itself, seized on the acousticus, whose irritation, by whatever source, is supposed to be the seat of this as yet not satisfactorily explained symptom to be daily met with in an aural clinic. These two—viz: the movements of the animals against the membrana tympani and the acoustic nerve—fully account for the above-mentioned annoying symptom, the roaring and singing; and even after the removal of the animals and the disappearance of the inflammation of the membrane, the slight, continued irritation of the nerve is explanatory of the remnant of the roaring still to a slight degree existing up to the day of his dismissal.

Also, in a medico-legal point of view, could the patient have made his case doubly interesting, and but for the perfect cure without permanent damage remaining as a result of the disease, he would have brought his combatant before the courts to answer for the trouble of body and mind caused possibly by his unlucky blow. In such investigations, medical men play an important part, and frequently for themselves an unpleasant part; hence it is necessary to know whether certain consequences are the results of certain injuries, what amount of injury can produce transitory and what lasting damage to the organ of hearing, and also what constitutes such a transitory or lasting damage, etc. The discussion of these points is not here intended, for it would lead us into another and in itself very extended chapter, but they are thrown out as a source of thought to those who may at any time be interested upon such a topic.

The punishment throughout Germany for such misdemeanor is fine or imprisonment, or both, in accordance with the resulting evil, as testified to and proven by the attending physician or medical expert. So, taking simply such a view, we at once see how absolutely necessary perfect treatment and exact observation of an affection is, which makes us, to some extent, the judgment as to the punishment or freedom of an individual.

Exactness and a simple statement of facts in medico-legal questions, is also the best protector for a physician against the rough handling to be sometimes met with at the hands of the legal profession.

Vienna, Austria, January, 1873.

---

Professional Risks.—It was stated in the memoir of M Guersant, read before the Surgical Society of Paris, that that eminent surgeon contracted syphilis through a wound in his finger received while operating upon a patient affected with that disease, and that his death was the ultimate result of the constitutional infection so produced.

